# Long-term neurodevelopmental outcomes in children with hypoplastic left heart syndrome

**DOI:** 10.1097/MS9.0000000000003731

**Published:** 2025-08-14

**Authors:** Kholoud Al Jebawi, Jamil Nasrallah, Waseem Sajjad, Areeba Inam, Mishaim Khan, Nourhan Kanso, Mira Al Shoufy, Nagham Naser Eldeen, Mirna N. Chahine

**Affiliations:** a Department of Medicine, Faculty of Medical Sciences, Lebanese University, Beirut, Lebanon; b Department of Life Sciences, Faculty of Sciences, Lebanese University, Beirut, Lebanon; c Department of Medicine, King Edward Medical University, Mayo Hospital, Lahore, Pakistan; d Department of Medicine, Ayub Medical College, Abbotabad, Pakistan; e Department of Medicine, FMH College of Medicine and Dentistry, Lahore, Pakistan; f Department of Biology, Faculty of Arts & Sciences, University of Balamand, Dekwaneh, Lebanon; g Foundation- Medical Research Institutes, Acharfieh, Beirut, Lebanon, Geneva, Switzerland

**Keywords:** cardiac surgery, congenital heart disease, hypoplastic left heart syndrome (HLHS), neurodevelopment, pediatric cardiology

## Abstract

**Background:**

Hypoplastic left heart syndrome (HLHS) is a severe congenital heart defect requiring complex surgical interventions, including the Norwood, Glenn, and Fontan procedures. This narrative review aims to explore the long-term neurodevelopmental outcomes of children with HLHS following surgical palliation with potential risk factors.

**Objectives:**

This review synthesizes current evidence on long-term ND outcomes in children with HLHS after surgical palliation and identifies key risk factors, assessment tools, and emerging neuroprotective strategies.

**Methods:**

A comprehensive literature review was conducted, focusing on the interplay of altered fetal circulation, impaired cerebral oxygenation, and extensive medical interventions on neurodevelopment. We conducted literature search of full-text, peer-reviewed English articles published between 2000 and 2024 using PubMed, Scopus, and Google Scholar with the terms “hypoplastic left heart syndrome,” “surgical palliation,” “neurodevelopmental outcomes,” and “children.”

**Results:**

Evidence indicates that children with HLHS face significant risks for cognitive, motor, behavioral, and adaptive impairments. Assessment tools such as the Bayley Scales of Infant and Toddler Development, MRI, and EEG are essential for early detection and ongoing monitoring.

**Discussion:**

A multidisciplinary approach involving pediatric cardiology, neurology, and developmental therapy is crucial for managing HLHS and addressing neurodevelopmental issues. Early intervention can improve outcomes, guided by diagnostic tools like MRI, EEG, and the Bayley Scales. There is a need for more research on neuroprotective strategies and perioperative management to reduce brain injury and enhance long-term developmental outcomes in children with HLHS.

**Conclusion:**

Children with HLHS face significant ND challenges due to a complex interplay of physiological and medical factors. A multidisciplinary approach with early intervention is critical. Emerging strategies, such as maternal hyperoxygenation, merit further investigation regarding their feasibility, cost, and ethical considerations.

## Introduction

Hypoplastic left heart syndrome (HLHS) is a critical congenital cardiac anomaly characterized by underdevelopment of the left heart structures, encompassing the left ventricle, mitral valve, aortic valve, and aorta^[1]^. This condition presents significant challenges in pediatric cardiac care, necessitating immediate and complex medical interventions. The prevalence of HLHS is estimated to be approximately 1 in 4344 live births, underscoring its significance within the spectrum of congenital heart diseases^[1,2]^. Early diagnosis is imperative for survival and is typically achieved through prenatal echocardiography or postnatal clinical assessment, with hallmark symptoms including cyanosis, lethargy, and impaired feeding capabilities^[1,3]^. Management of HLHS requires a staged surgical palliation approach, consisting of the Norwood procedure shortly after birth, followed by the Glenn and Fontan procedures at subsequent developmental stages^[4,5]^. These interventions are designed to reconfigure the heart’s circulation, compensating for the underdeveloped left heart structures and improving systemic oxygen delivery^[5]^. The motivation for this review stems from the imperative need to systematically evaluate the long-term neurodevelopmental outcomes in children with HLHS following these surgical interventions. Given the critical nature of these interventions and the vulnerability of the patient population, there has been growing interest in understanding the long-term outcomes of these surgical palliations, particularly concerning neurodevelopmental trajectories.

Children with HLHS are at a heightened risk for neurodevelopmental impairments due to factors such as reduced cerebral oxygen delivery, the potential impact of cardiopulmonary bypass during surgery, and the cumulative effects of multiple hospitalizations and medical interventions throughout early childhood^[6]^. The exploration of long-term neurodevelopmental outcomes in children with HLHS who have undergone surgical palliation is crucial for several reasons. First, it provides insights into the efficacy of current surgical practices and their implications for the child’s cognitive, motor, communicative, and socio-emotional development^[7]^. Second, it informs clinical practices and interventions aimed at optimizing developmental outcomes for this vulnerable population. Lastly, it guides families and educators in supporting the unique needs of children with HLHS as they grow and mature^[6–8]^. Prenatal detection and early intervention, which detects abnormal brain structures during pregnancy and enables the planning of early interventions, are comprehensive strategies and interventions to improve these outcomes of these patients. Additionally, it is crucial to start neurodevelopmental therapy at birth and provide developmental support in pediatric cardiac critical care units^[5,8]^. Furthermore, for newborns at high risk of delayed development, regular postnatal care guarantees early identification and the start of appropriate therapies^[6–8]^.

This review aims to synthesize the current body of research on the neurodevelopmental outcomes of children with HLHS post-surgical palliation, focusing on studies that have followed these children through their childhood and beyond. By examining the scope and quality of existing research, this review seeks to identify patterns in neurodevelopmental outcomes, potential risk factors or developmental delays, and areas requiring further investigation. The ultimate goal is to contribute to a comprehensive understanding that supports the ongoing refinement of treatment protocols, enhances patient care strategies, and facilitates the development of supportive educational and social services tailored to the needs of children with HLHS and their families.

## Methodology

This narrative review provides a comprehensive methodology for determining the neurodevelopmental outcome of children with HLHS, including insights into pathophysiological origins, clinical repercussions, and potential treatment methods. The inclusion criteria were full-text papers in English published between 2000 and 2024. This timeline was chosen to suit the most recent advancements. A thorough literature search was carried out using the PubMed, Scopus, and Google Scholar databases. The search phrases were “hypo plastic left heart syndrome,” “surgical palliation,” “neurodevelopmental outcomes,” and “children.” We meticulously followed our exclusion criteria to maximize the chance of delivering trustworthy and reproducible data while reducing potential bias. The exclusion criteria excluded case reports, non-English studies without results, conference papers, posters, and unpublished or non-peer-reviewed research. Furthermore, our study employed a meshwork of around 425 papers, of which 211 were examined and included in our research. Table 1 provides a clear description of the approach used.

**HIGHLIGHTS**

- Children with hypoplastic left heart syndrome (HLHS) face elevated risks for cognitive, motor, behavioral, and adaptive delays after surgical palliation.
- Altered fetal circulation and impaired cerebral oxygenation are key contributors to neurodevelopmental challenges in HLHS patients.
- Tools like the Bayley Scales, MRI, and EEG are critical for early diagnosis and ongoing assessment of developmental issues in HLHS.
- A multidisciplinary care approach, involving cardiology, neurology, and developmental therapy, is essential for optimizing outcomes in HLHS children.
- Further research into neuroprotective strategies during perioperative care is needed to improve the long-term neurodevelopment of children with HLHS.

**Table 1:** Summary of the methodology

Methodology steps	Description
Literature search	PubMed, Scopus, and Google Scholar databases
Inclusion criteria	Full-text articles in English published between 2000 and 2024 that focused on hypoplastic left heart syndrome, surgical palliation, neurodevelopmental outcomes, and children
Exclusion criteria	Case reports, Studies not written in English, Studies that do not report outcomes, Conference papers, Posters, Unpublished and non-peer-reviewed studies
Search terms	Keywords such as “hypoplastic left heart syndrome,” “surgical palliation,” “neurodevelopmental outcomes,” and “children”

Demographic diversity: We incorporated research from all over the world based on our review. Race, ethnicity, and socioeconomic status were not widely reported in studies, and many only included age and gender as demographic information. Comprehensive information on other demographic factors was limited, despite the fact that multiple studies reported a slight male predominance. The findings on neurodevelopmental outcomes may not be as broadly applicable to more diverse populations as they could be due to this relative homogeneity and the underreporting of important demographic variables. To fill this knowledge gap, we advise that future studies methodically gather and present comprehensive demographic data in order to gain a better understanding of the potential effects of these variables on the outcomes of children with HLHS.

## Procedures for HLHS management

### The Norwood procedure: foundation for HLHS management

The Norwood procedure is crucial in managing HLHS as it focuses on creating a stable systemic circulation, forming the foundation of treatment for this condition^[9]^. Innovations in this procedure, particularly the debate between utilizing a right ventricle-pulmonary artery (RV-PA) conduit versus a modified Blalock-Taussig (BT) shunt, have significantly influenced postoperative management and patient outcomes. Studies indicate that while the RV-PA conduit offers improved early postoperative hemodynamics, long-term outcomes between the two approaches are comparable^[3,9,10]^. The Norwood procedure involves several key surgical interventions. Firstly, since the main pulmonary artery and the aorta are connected, the pulmonary artery branches are separated to construct a new aorta, which is then connected to the right ventricle through a shunt^[6,11,12]^. A critical component of this procedure is the establishment of a stable source of pulmonary blood flow, which can be achieved through either a modified Blalock-Taussig (BT) shunt or a right ventricle-pulmonary artery (RV-PA) conduit^[11–13]^. Despite its critical role in the management of HLHS, the Norwood procedure is associated with significant risks and complications. These can include issues related to the shunt, such as blockage or excessive blood flow to the lungs and brain, as well as potential problems with the heart’s rhythm and function^[6,12,14]^.

### The Glenn procedure

The Glenn procedure, formally known as the bidirectional Glenn shunt, represents a pivotal phase in the staged palliation for children with single-ventricle congenital heart defects, such as HLHS^[4,15]^. This procedure is typically performed when the child is between 4 and 6 months old, following the initial Norwood procedure. The primary aim of the Glenn procedure is to alleviate some of the workload on the heart by rerouting blood flow directly to the lungs, bypassing the heart^[11,14,15]^. The Glenn procedure involves connecting the superior vena cava (SVC) directly to the pulmonary arteries^[16]^. This surgical modification allows oxygen-poor blood from the upper half of the body to flow directly into the lungs without passing through the heart, thereby reducing the volume of blood the single ventricle must pump and improving oxygenation levels^[4,16]^. By redirecting blood flow directly to the lungs, the procedure reduces the pressure and volume load on the single functional ventricle^[16,17]^. Glenn procedure has been shown to significantly improve the quality of life and survival rates for children with HLHS, allowing most to reach the final stage of palliation, the Fontan procedure^[18–21]^.

### Fontan procedure

The third surgical procedure, called the Fontan procedure, is typically conducted around 3 to 4 years after the Glenn procedure^[22]^. It represents the last step in the surgical treatment, aiming to achieve complete separation of systemic arterial and venous blood flow. This procedure redirects the flow of blood from the inferior vena cava directly to the pulmonary artery, bypassing the heart^[23]^.The ultimate goal of this palliative surgical therapy is to establish what is known as full Fontan circulation, where all systemic venous blood enters the pulmonary circulation rather than returning to the right atrium^[24]^. The surgery can be carried out through two main methods: first, by constructing a lateral tunnel using a patch to link the entrances of the inferior and superior vena cava, and second, by adapting the initial Fontan technique through the use of an extra-cardiac conduit, typically a vascular prosthesis, to connect the inferior vena cava directly to the pulmonary artery^[25–27]^.

## Neurodevelopmental challenges in HLHS

### Factors that may affect brain development and function in HLHS

Children with HLHS meet significant neurodevelopmental challenges due to complex interaction of physiological anomalies that start even before birth^[28]^. These challenges are fundamentally due to altered fetal circulation, impaired cerebral oxygenation, reduced nutritional supply and neurological injury during medical interventions^[28,29]^.

#### Altered fetal circulation

HLHS is a severe congenital condition denoted by a negatively underdeveloped left ventricle^[30,31]^, leading to marked changes in both fetal and postnatal circulation^[29]^. In fetuses with HLHS, the left ventricle is exposed to a 65% depletion in end-diastolic volume principally due to mitral stenosis^[30]^, necessitating that the right ventricle assumes full responsibility for systemic circulation^[32]^. This is obvious before birth with the abnormal flow patterns such as reversed shunting at the foramen ovale and primary systemic circulation bypassing the left lung via the ductus arteriosus, leading to diminished pulmonary perfusion and right ventricular volume overload^[32]^. Postnatally, the challenges raise and the 4D patient-specific ultrasound scans show almost the same hemodynamics between normal and HLHS right ventricles as the foramen ovale and ductus arteriosus close after birth^[32,33]^.

Simultaneously, HLHS is often associated with aortic stenosis, contributing to reversed flow in the aortic arch and left to right shunting at the atrial level^[34]^. These circulatory anomalies are verified by decreased systemic and superior vena cava flow in neonates with HLHS compared to controls, along with increased resistive indices in the middle cerebral and celiac arteries, showing impaired cerebral and systemic blood flow^[35]^. Unfortunately, studies showed that even interventions post-birth, including surgeries performed to bypass the dysfunctional left ventricle, further complicate the heart’s dynamics, as the right ventricle is insufficiently loaded due to obstructions caused by these surgeries at the pulmonary circulation and increased clogged in systemic veins, leading to potential congestive heart failure and altered circulation^[32,36]^.

#### Impaired cerebral oxygenation

Impaired cerebral oxygenation in children with HLHS significantly impacts brain development and function. Recent studies have provided comprehensive insights into how reduced oxygen delivery to the brain during both fetal and postnatal periods affects these children.

Fetuses with HLHS experience reduced cerebral blood flow and oxygen delivery. This impairment is primarily due to the abnormal cardiac structure and the retrograde cerebral perfusion through a patent ductus arteriosus, which diminishes the oxygenation of blood reaching the brain. Simulation studies have shown that the oxygen saturation of cerebral blood flow in newborns with HLHS is significantly lower than in normal fetuses, leading to reduced cerebral oxygenation^[37]^.

The impact of this reduced oxygenation is evident in the brain size of affected fetuses. Studies utilizing MRI have demonstrated that reduced fetal cerebral oxygen consumption is associated with smaller brain size in fetuses with congenital heart disease, including HLHS. This correlation suggests that lower oxygen delivery and consumption directly impair brain growth, leading to decreased brain volume and developmental deficits^[38]^.

Impaired cerebral oxygen delivery also adversely affects cortical development in infants with congenital heart disease. Research indicates that these infants exhibit reduced cortical gray matter volume and gyrification index, which are critical indicators of brain development. These findings highlight the compromised brain development resulting from inadequate oxygen supply^[39]^.

Moreover, neurodevelopmental outcomes in children with HLHS are significantly influenced by preoperative cerebral oxygen saturation levels. Studies have found that lower preoperative oxygen levels are associated with lower IQ and cognitive function scores. This suggests that the degree of cerebral oxygenation before surgical intervention plays a crucial role in determining cognitive outcomes in these children^[6]^.

There is promising evidence that maternal hyperoxygenation can increase cerebral blood oxygenation in fetuses with HLHS. This intervention has the potential to improve fetal brain oxygenation and development, offering a possible strategy to mitigate the adverse effects of impaired cerebral oxygenation^[40]^. Despite the potential benefits of maternal hyperoxygenation in improving fetal oxygenation and possibly reducing neurodevelopmental risks, its incorporation into standard clinical practice necessitates a thorough assessment of its viability, cost-effectiveness, and ethical implications^[37,39]^. In order to ascertain whether the strategy can be applied successfully and safely, recent research highlights the importance of conducting a thorough evaluation that takes into account economic factors, the need for specialized infrastructure and trained personnel, and maternal-fetal risks^[6]^. To protect the health of the mother and fetus, ethical examination is also necessary to guarantee informed consent, fair access, and a balanced risk-benefit profile. Large-scale research and interdisciplinary assessments are therefore required to confirm that the long-term advantages outweigh the possible risks and the investment before broad clinical adoption^[40]^.

#### Reduced nutritional supply

Due to impaired systemic circulation and decreased gastrointestinal perfusion, the nutritional supply is reduced, limiting nutrient absorption and delivery to the brain, which critically impacts brain growth and development, as well as early learning and cognitive development^[41,42]^. Essential nutrients such as protein, omega-3 fatty acids, iron, iodine, zinc, and choline play crucial roles in neurodevelopmental processes like neuron proliferation, myelination, and synaptogenesis^[41]^. For instance, iron deficiency during early infancy has been linked to impaired attention, memory, and behavior, and is associated with lower cognitive indices and poorer school performance later in life^[43]^. Moreover, long-chain polyunsaturated fatty acids (PUFAs), for example, are critical for brain tissue development and deficiencies in these PUFAs, as well as vitamins D, B12, and folate, can lead to significant cognitive and behavioral deficits^[44]^.

In Addition, studies have shown also that maternal multiple micronutrient supplementation during pregnancy has long-term benefits on cognitive development, including improved procedural memory and general intellectual ability in children^[45]^. Similarly, maternal nutritional status during pregnancy is a predictor of offspring cognitive function, highlighting the importance of adequate maternal nutrition for optimal child development^[46]^. Reduction in this nutritional supply due to impaired systemic circulation is associated with lower cognitive skills and school performance^[47]^.

Furthermore, early nutrition influences epigenetic mechanisms that regulate gene expression, impacting brain development and long-term health outcomes. For example, inadequate nutrition can lead to aberrant DNA methylation patterns that are linked to neurodevelopmental and cognitive impairments^[48]^.

#### Neurological injuries

HLHS can significantly affect brain development and function also by causing neurological injuries in children through several mechanisms related to impaired oxygen delivery and circulation, surgical interventions, and the resulting metabolic and inflammatory responses.

For example, HLHS can cause hypoxic-ischemic (HI) injury due to diminished cerebral blood flow and oxygen content^[40]^, resulting from both the congenital heart defect itself and the necessary surgical interventions^[49,50]^, which triggers a cascade of cellular and biochemical pathways that lead to neuronal injury, including inflammation and neuroimmune dysregulation, disrupting the normal developmental processes of the brain, and leading to long-term cognitive and neurological disabilities^[51]^.

Moreover, hypoxic brain injury in children can lead to significant psychomotor developmental delays, which can result in hyperactivity, delayed speech and cognitive development, and motor function impairments^[52]^.

Furthermore, inflammation is a key pathway in the progression of neuronal injury after HI. The activation of microglia and the release of proinflammatory cytokines can exacerbate this brain injury and damage, further impairing neurodevelopmental processes^[51]^.

Besides, altered cerebral circulation and oxygen content can cause perinatal brain injuries which are associated with an increased prevalence of neurological, behavioral, and psychiatric problems in later life. Studies have shown that children who experience perinatal brain injury are at higher risk for long-term cognitive impairments, including difficulties with attention, memory, and executive function^[53]^.

Additionally, children with HLHS who undergo surgical interventions, such as the hybrid procedure, show varying neurodevelopmental outcomes. While some studies report favorable cognitive, language, and motor development at 2–3 years of age, others highlight the presence of brain abnormalities, such as reduced white matter volumes and increased cerebrospinal fluid (CSF) volumes, which can influence long-term neurodevelopmental outcomes^[54]^.

### Neurodevelopmental impairments in children with HLHS

Children who have HLHS suffer from severe neurodevelopmental problems, such as defects in their cognitive, motor, behavioral, and adaptive abilities. A combination of neurological injury, decreased nutritional supply, impaired cerebral oxygenation, and altered fetal circulation results in these defects^[54]^. According to numerous studies, children with HLHS frequently have cognitive impairments, including issues with executive function, attention, memory, and processing speed. These cognitive impairments can have a significant impact on daily functioning and academic performance^[55,56]^. The underdevelopment of the left heart in HLHS interferes with normal fetal circulation, which is essential for delivering oxygen and nutrients during critical phases of brain development. This leads to neurological damage that occurs even before birth due to impaired cerebral oxygenation and this altered circulatory pattern^[54,56]^.

Children with HLHS also often have mild to severe motor impairments that impact their activities of daily living and participation in physical activities^[57]^. These motor deficiencies, which can impede general physical development and independence, can include problems with balance, coordination, and fine motor skills^[58,59]^. Children with HLHS are also likely to experience behavioral and mental health issues. These kids are more likely to experience anxiety, depression, and attention deficit hyperactivity disorder (ADHD)^[60,61]^. These conditions can further complicate adaptive behaviors and social interactions, making it more difficult for kids with HLHS to build and sustain relationships and participate in school and community activities^[62]^.

These factors work together to highlight the importance of early detection and intervention. Technological developments in neuroimaging and longitudinal neurodevelopmental evaluations have made it possible for physicians to detect these deficiencies earlier. Targeted interventions, such as cognitive therapies and motor and behavioral support, can be implemented with early detection to help mitigate long-term impairments and enhance overall quality of life^[55,62]^. Neurodevelopmental assessments, focused treatments, and ongoing monitoring are all essential components of a comprehensive approach that can help children with HLHS achieve their best results and guarantee they get the help they require to realize their full potential.

## Neurodevelopmental assessment and intervention in HLHS

### Methods and tools for assessing neurodevelopmental outcomes in HLHS

As a developmental evaluation, the BSID-II and BSID-III versions of the Bayley Scales of Infant and Toddler Development are applied. While BSID-III consists of cognitive, language, and motor components, BSID-II consists of the Mental Developmental Index (MDI) and Psychomotor Developmental Index (PDI) (64). Brain injury and neurodevelopmental outcomes in HLHS are assessed using perioperative brain MRI (64).To evaluate brain volumes, cerebral MRI and transcranial Doppler cerebral blood flow (CBF) from the middle cerebral artery were analyzed from the results of cerebral imaging (65).To assess behavioral and executive functioning problems, questionnaires like the Sure Start Language Measure (SSLM), Strengths and Difficulties Questionnaire (SDQ) and the Behavior Rating Inventory of Executive Function (BRIEF) are used (66,67). A noninvasive method for assessing somatic oxygen saturation (Sso2) and regional cerebral oxygen saturation (Sco2) is near-infrared spectroscopy (NIRS) (68). For the purpose of early neurodevelopmental risk identification in 2-year-old infants with hypoxic ischemic encephalopathy, the interpretation of magnetic resonance imaging (MRI), general movement assessment (GMA), and the Hammersmith Infant Neurological Examination (HINE) may be the gold standard (69). When an infant has congenital heart disease, the neonatal electroencephalogram (EEG) is a sensitive way to assess brain damage and function (70). Additionally, Child Behavior Checklist (CBCL) and Vineland Adaptive Behavioral Scales (VABS) evaluate the developmental outcomes (71). The primary demographics of the caregiver and the child with HLHS are investigated using three validated questionnaires: the Pediatric Quality of Life Inventory (PedsQL), the Parenting Stress Index-Short Form (PSI-SF), and the Pediatric Inventory for Parents (PIP) (72) (Table 2).

**Table 2:** Developmental evaluation tools recap

Tool	Purpose	Reference
BSID-II (Bayley Scales of Infant and Toddler Development II)	Measures Mental Developmental Index (MDI) and Psychomotor Developmental Index (PDI)	^[63]^
BSID-III (Bayley Scales of Infant and Toddler Development III)	Assesses cognitive, language, and motor development	^[63]^
Perioperative brain MRI	Evaluates brain injury and neurodevelopmental outcomes	^[63]^
Cerebral MRI and Transcranial Doppler CBF	Analyzes brain volumes and cerebral blood flow from the middle cerebral artery	^[64]^
Sure Start Language Measure (SSLM)	Assesses language development	^[65]^
Strengths and Difficulties Questionnaire (SDQ)	Evaluates behavioral difficulties	^[65]^
Behavior Rating Inventory of Executive Function (BRIEF)	Assesses executive functioning problems	^[66]^
Near-infrared Spectroscopy (NIRS)	Measures somatic oxygen saturation (Sso2) and regional cerebral oxygen saturation (Sco2)	^[67]^
MRI, General Movement Assessment (GMA), Hammersmith Infant Neurological Examination (HINE)	Identifies early neurodevelopmental risks in infants with hypoxic ischemic encephalopathy	^[68]^
Neonatal Electroencephalogram (EEG)	Assesses brain damage and function in infants with congenital heart disease	^[69]^
Child Behavior Checklist (CBCL)	Evaluates developmental outcomes and behavior	^[70]^
Vineland Adaptive Behavioral Scales (VABS)	Assesses adaptive behavior and development	^[70]^
Pediatric Quality of Life Inventory (PedsQL)	Evaluates quality of life in pediatric patients	^[71]^
Parenting Stress Index-Short Form (PSI-SF)	Measures parenting stress levels	^[71]^
Pediatric Inventory for Parents (PIP)	Assesses parental experiences with their child’s illness	^[71]^

### Strengths and limitations of different assessment methods

Standardized tests such as the Bayley Scales of Infant and Toddler Development (Bayley-III) are widely used for evaluating cognitive, language, and motor development in young children. The Bayley-III is praised for its comprehensive assessment capabilities but has limitations in sensitivity, particularly in detecting mild impairments in cognitive and communication domains^[63]^. Moreover, questionnaires like the Strengths and Difficulties Questionnaire (SDQ) and the Behavior Rating Inventory of Executive Function (BRIEF) are beneficial for identifying behavioral and executive functioning issues and are valued for their ease of use and ability to provide comprehensive behavioral profiles. However, their reliance on parent or teacher reports can introduce bias and variability^[64]^.

Furthermore, neuroimaging techniques, including MRI and amplitude-integrated EEG (aEEG), are critical for detecting brain abnormalities and predicting long-term outcomes. MRI offers detailed anatomical information but can be challenging to perform in very young or medically unstable children, whereas aEEG is less invasive and provides real-time brain function data but is limited in its ability to localize specific brain injuries, and using a combination of assessment tools to provide a comprehensive evaluation of neurodevelopmental outcomes is a must^[28]^.

As well, tools like the General Movement Assessment (GMA) and the Hammersmith Infant Neurological Examination (HINE) are particularly useful for early identification of neurodevelopmental delays and cerebral palsy, implying the need for accurate and reliable use, which often requires specialized training^[65]^. Also, the sensitivity and specificity values for determining the psychomotor developmental index (PDI), mental developmental index (MDI), and CP diagnosis indicate that while these tools are generally reliable, there is some variability. For example, the specificity of HINE for determining MDI is relatively low (50%), which suggests that there might be a higher rate of false positives when using HINE for this purpose^[65]^. These are summarized in the table below (Table 3).

**Table 3:** The table summarizing the strengths and weaknesses of the assessment tools

Tool	Strengths	Weaknesses
Bayley Scales of Infant and Toddler Development (Bayley-III)	Comprehensive assessment of cognitive, language, and motor development	Limited sensitivity in detecting mild cognitive and communication impairments
Strengths and Difficulties Questionnaire (SDQ)	Easy to use, provides comprehensive behavioral profiles	Reliance on parent/teacher reports introduces bias and variability
Behavior Rating Inventory of Executive Function (BRIEF)	Identifies behavioral and executive functioning issues, easy to administer	Subject to variability and bias due to reliance on parent/teacher reports
Magnetic Resonance Imaging (MRI)	Provides detailed anatomical brain information	Challenging to perform in very young or medically unstable children
Amplitude-Integrated EEG (aEEG)	Real-time brain function data, less invasive	Limited ability to localize specific brain injuries
General Movement Assessment (GMA)	Useful for early identification of neurodevelopmental delays and cerebral palsy	Requires specialized training for accurate use
Hammersmith Infant Neurological Examination (HINE)	Accurate in identifying early neurodevelopmental delays and CP, specialized for motor assessments	Lower specificity for determining mental developmental index (MDI), higher false positive rate

### Optimal timing and frequency of neurodevelopmental outcomes of HLHS and factors influencing the results

Assessments should begin in infancy, as early as 2 months post-surgery, using tools like the Test of Infant Motor Performance (TIMP) and continue at 4 and 6 months using the Bayley Scales of Infant and Toddler Development (Bayley-III)^[66]^. Longitudinal follow-ups are essential also, particularly at key developmental milestones such as 12 months, 24 months, and early school age, to monitor ongoing cognitive, motor, and behavioral development^[67]^.

In addition to timing and frequency, several factors significantly influence the results of neurodevelopmental assessments in children with HLHS, including age, stage of palliation, and comorbidities. The age of the child at the time of assessment is crucial, as developmental trajectories can vary significantly across different age groups, necessitating age-specific evaluation tools and benchmarks^[54,68]^. The stage of surgical palliation also plays a vital role, as children at different stages (e.g., Hybrid Stage I or following the Norwood procedure) exhibit distinct neurodevelopmental patterns. Early assessments post-surgery is critical for identifying developmental delays and tailoring intervention strategies accordingly^[66]^. Additionally, comorbidities such as genetic anomalies and other medical conditions can exacerbate developmental challenges. For instance, children with HLHS and additional genetic disorders often present with more severe neurodevelopmental impairments, highlighting the need for comprehensive and multidisciplinary assessment approaches^[69]^. Hemodynamic factors, such as elevated central venous and atrial filling pressures, are also associated with poorer neurodevelopmental outcomes, indicating the importance of considering cardiovascular health in these assessments^[70]^. Overall, these factors underscore the complexity of neurodevelopmental assessments in HLHS, necessitating a multifaceted and dynamic approach to effectively monitor and support affected children.

### Recommendations for improving neurodevelopmental outcomes in HLHS

**Figure 1.**
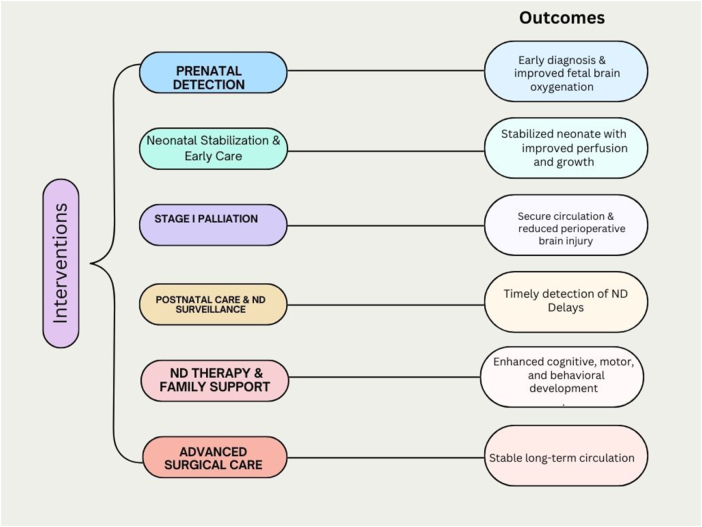
Comprehensive interventions to improve neurodevelopmental outcomes in HLHS patients.

HLHS patients are at a higher risk of adverse neurodevelopmental outcomes due to abnormalities in fetal circulation, changes in cerebral blood flow, and neurological injury^[29]^. Comprehensive strategies and interventions to improve these outcomes include prenatal detection and early intervention, which allows for the planning of early interventions by identifying abnormal brain structures during pregnancy^[29]^. Also, developmental support in pediatric cardiac critical care units and the initiation of neurodevelopmental therapy at birth are critical measures^[29,71]^. Moreover, consistent postnatal care ensures early identification and commencement of relevant therapies for newborns at high risk of delayed development^[72]^. Neuroprotective interventions such as developmental supportive care, including massage, kangaroo care, and cue-based feeding, promote adequate neurodevelopment in survivors of congenital cardiac disease also (2). Specific pedagogic and therapeutic interventions are also essential to aid the development of learning strategies, helping manage the described cognitive difficulties^[56]^ (Fig. 1). Furthermore, advances in medical care, particularly surgical procedures and perioperative care, have significantly improved the survival rates of children with functional single ventricles^[73–75]^. Additionally, the quality of family relationships and patient care are crucial for the overall well-being of HLHS patients, despite being beyond practitioners’ control^[73]^. Some of the interventions and outcomes have been explained in Fig. 1.

## Discussion

This narrative review aimed to explore the long-term neurodevelopmental outcomes of children with HLHS following surgical palliation. The main findings indicate that children with HLHS are at significant risk for various neurodevelopmental impairments, including cognitive, motor, behavioral, and adaptive deficits. These impairments are attributed to altered fetal circulation, impaired cerebral oxygenation, reduced nutritional supply, and the cumulative impact of multiple surgical interventions^[54]^.

Moreover, the findings of this review have several practical and clinical implications for the management and care of HLHS patients such as implementing routine use of neurodevelopmental assessment tools, such as the Bayley Scales of Infant and Toddler Development, MRI, and EEG, which can help in early identification of developmental delays and guide timely interventions^[76,77]^. Also, implementing developmental supportive care practices, such as massage, kangaroo care, and cue-based feeding, in pediatric cardiac critical care units can enhance neurodevelopment in infants with HLHS^[2]^. This condition can also be exacerbated by risk factors such as infections, neonatal encephalopathy, and prematurity. Improving long-term results requires early, thorough follow-up with age-appropriate screening in order to facilitate timely intervention. It is crucial to conduct early tests within the first few months following surgery using MRI, EEG, and developmental scales^[71,72,77]^. Moreover, improving results requires the use of efficient intervention techniques, such as developmental supportive care, neurodevelopmental therapies, and prenatal detection.^[29,71]^.

Long-term follow-up should include periodic Neurodevelopmental assessments and tailored interventions. Individualized therapies, like speech, occupational, and physical therapy, are essential for meeting each child’s particular developmental needs. These specialized treatments support the development of effective communication skills, improved motor skills, and improved sensory processing. These interventions can support increased independence and quality of life by concentrating on personal strengths and challenges^[56,71]^. Regular evaluations with standardized screening instruments enable clinicians to track a child’s development over time, identify developmental delays early, and modify intervention tactics as necessary. This continuous assessment makes sure that any new problems are dealt with right away, increasing the program’s overall efficacy. In addition to the strategies discussed, several approaches can further enhance neurodevelopmental outcomes for children with HLHS. For instance, a comprehensive, multidisciplinary approach involving cardiologists, neurologists, developmental specialists, and other healthcare providers is essential for addressing the diverse needs of HLHS patients. Collaborative care can improve overall management and outcomes. Furthermore, providing education and support to parents is crucial, as family relationships and patient care quality significantly influence neurodevelopmental outcomes^[73]^. Educating parents about the importance of early intervention and continuous monitoring can empower them to advocate for their child’s needs. Additionally, integrating neurodevelopmental therapies, such as physical, occupational, and speech therapy, into the standard care protocol for HLHS patients can address cognitive, motor, and behavioral deficits, promoting better developmental outcomes^[56]^.

Limitations of existing evidence: Despite the advancements in understanding neurodevelopmental outcomes in children with HLHS, several gaps and limitations remain. One major limitation is the heterogeneity of study designs and methodologies, which complicates the comparison and synthesis of results across studies. Many studies rely on small sample sizes and single-center data, limiting the generalizability of their findings^[78]^. Additionally, there is a lack of longitudinal studies that track neurodevelopmental outcomes from infancy through adolescence and adulthood. Such studies are crucial for understanding the long-term impacts of HLHS and its treatments^[79]^. Another significant gap is the limited research on the potential benefits of emerging neuroprotective interventions, such as maternal hyperoxygenation, which could improve fetal brain development. Differences in surgical techniques, perioperative management, and neurodevelopmental assessment tools contribute to inconsistent findings across studies. Patient-specific factors, including genetic variations and socioeconomic influences, further add to this heterogeneity. A summary of the main findings from the major studies on early interventions, nutritional impact, and surgical outcomes is provided in Table 1. This table identifies areas with limited or inconsistent evidence as well as consistent findings. We have also included suggestions for further study in this field.

Future recommendations: Future research should focus on large-scale, multicenter longitudinal studies to provide a more comprehensive understanding of neurodevelopmental trajectories in children with HLHS. Investigating the efficacy of new neuroprotective strategies and their implementation in clinical practice is also necessary. Long-term follow-up should include periodic neurodevelopmental assessments and tailored interventions targeting physical, occupational, and speech therapies. Some of these recommendations have been summarized in Table 4.

**Table 4:** Future recommendations and current gaps in early interventions, nutritional impact, and surgical outcomes in current literature

Area of focus	Current gaps	Priority areas for future research
Surgical outcomes	There is little long-term follow-up data and early outcome variability between BT shunt and RV-PA conduit	Longitudinal, multicenter studies evaluating surgical methods and their effects on neurodevelopment over time
Nutritional impact	Limited evidence regarding how certain dietary deficiencies affect neurodevelopment	Examine early nutritional support procedures and focused nutritional interventions
Early interventions	Inadequate long-term efficacy data and inconsistent intervention protocols	Assess long-term cognitive, motor, and behavioral results and standardize early neurodevelopmental interventions

## Conclusion

This review highlights the significance of managing HLHS holistically, with the goal of enhancing the general neurodevelopmental trajectories and promoting positive outcomes of impacted children and their families. Children with HLHS face substantial neurodevelopmental challenges due to a complex interplay of physiological and medical factors. Comprehensive, multidisciplinary approaches involving early detection, continuous monitoring, and tailored interventions are necessary to optimize long-term developmental outcomes. Advancements in surgical techniques and perioperative care have improved survival rates, but ongoing support and personalized therapeutic strategies are vital for enhancing the quality of life for children with HLHS. While public health initiatives should increase access to specialized CHD care, integrate neurodevelopmental tracking into national registries, and fortify parental education programs to enhance long-term outcomes, researchers should concentrate on multicenter, longitudinal studies to improve intervention protocols.

## Data Availability

Not applicable.

## Research registration unique identifying number (UIN)

No registration was required for this review.
